# Psychiatric treatment outcomes of travelers admitted to a psychiatric hospital: a retrospective analysis

**DOI:** 10.1186/s40794-024-00244-x

**Published:** 2025-04-30

**Authors:** Achim Burrer, Tobias R. Spiller, Jose Marie Koussemou, Georgios Schoretsanitis, Philipp Homan, Steffi Weidt, Erich Seifritz, Stefan Vetter, Stephan T. Egger

**Affiliations:** 1 Department of Adult Psychiatry and Psychotherapy, Psychiatric Hospital, University of Zurich, Zurich, Switzerland; 2 Faculty of Medicine, University of Ulm, Ulm, Germany; 3 Psychiatry Research, Northwell Health, The Zucker Hillside Hospital, 75-59 263rd Street, Glen Oaks, NY 11004 USA; 4 Zucker School of Medicine at Hofstra/Northwell, Hempstead, NY 11549 USA; 5 Neuroscience Center Zurich, University of Zurich, Zurich, Switzerland; 6 Psychiatric Services St. Gallen, Wil, SG Switzerland

**Keywords:** Traveler, Tourist, Psychiatric, Inpatient, Repatriation, Travel-related psychosis

## Abstract

**Background:**

Travel-related psychiatric disorders range from anxiety disorders to mood disorders, substance abuse, and psychosis. Various travel-associated factors such as dehydration, time shifts, changes in social structures or stress factors are discussed for these disorders. There is a lack of knowledge concerning the quality and outcome of psychiatric treatment in travelers hospitalized abroad. This study is the first to compare outcome of treatment in psychiatric travelers to domestic patients.

**Methods:**

We analyzed electronic health records of travelers in the Psychiatric University Hospital Zurich from January 2013 to December 2020. Each traveler was matched with one Swiss national and one migrant using propensity score matching.

**Results:**

Travelers showed inferior CGI-I scores at discharge (*F*(2,969) = 5.72; *p* = 0.003). The length of stay was shorter (*F*(2,969) = 38.74:*p* < 0.001) for travelers (9.69 ± 14.31) than for Swiss nationals (24.69 ± 29.42) and migrants (24.74 ± 28.62). The transfer rate to another hospital was higher (*X*^2^(2,972) = 50.85: *p* < 0.001) for travelers (79, 29.4%) than for Swiss nationals (25, 7.7%) or migrants (26, 8.0%).

**Conclusions:**

Psychiatric treatments of hospitalized travelers showed a lower symptom improvement while presenting a more severe overall condition at discharge. Length of stay was shorter compared to domestic patients. Admission of travelers was initiated involuntarily more frequently. This most closely reflects the theory that travelers are typically hospitalized in severe emergencies and are promptly discharged or repatriated after an initial treatment response has been achieved.

## Introduction

Travel, whether for business or pleasure, often signifies a break from routine, affording novel experiences and encounters. For some individuals, the disruption of habitual patterns and the exposure to unfamiliar environments can precipitate significant psychological distress, leading to the onset or exacerbation of psychiatric disorders (pathogenic travel) [1]. In terms of psychosis besides travel-related factors like cultural shock, insomnia, jet lag, dehydration, time zone switched exacerbations [2] and travel-induced psychosis in elderly [3], some destinations themselves are described to be psychosis-inducing [4] the most well-known of which is Jerusalem syndrome [5]. There is also travel that can occur as a pathological behavior (e.g. dissociative fugue or psychotic-motivated escape) as part of a psychiatric disorder such as a pre-existing psychotic illness motivating the patient to travel (pathologic travel) [4]. In this context psychotic disorders frequently manifest through ambulatory behavior patterns, particularly pathologic travel - defined as voyages undertaken within a delusional framework that directly correlate with the patient’s psychotic state [6]. Prior research examining patients admitted to psychiatric emergency unit in Paris has documented a notable prevalence of this phenomenon [7].

Psychiatric disorders related to travel span a spectrum of conditions, encompassing anxiety disorders such as acute stress reaction and panic disorder, mood disorders, substance-related disorders, and psychosis [8, 9].

In addition to the fact that psychiatric admissions can be explained by pathogenic or pathologic travel, the fact that the frequency of psychiatric admissions is influenced by external events such as natural disasters or pandemics is also described [10].

Overall, mental disorders are a leading cause of illness among travelers. A recent review states that among 19.7 out of 100,000 travelers require a form of psychiatric treatment and 8.4 require hospitalization [4]. Additionally, 2.4–3.1% of all in-flight emergencies can be categorized as psychiatric [11, 12]. Psychiatric illness furthermore is among the most frequent causes for medical evacuations [13] among which psychotic disorders make up a part of 10–20% [14]. This field of inquiry, focusing on travel-related psychiatric disorders leading to hospitalization, remains relatively underexplored [15], yet has substantial implications for the individual patient, public health and clinical psychiatry [16].

Repatriation of mentally ill is a procedure already described in British India in the 19th century [17] and is nowadays a standard procedure in psychiatric patients hospitalized abroad [18]. Psychiatric repatriation should thereby follow a standardized repatriation process [18] as applied at our institution in line with a way of transport following international standards [19]. While in Switzerland repatriation is paid for by the Swiss state, repatriation is often limited due to costs and insurance policies in other countries [1].

Despite the prevalence of psychiatric illness in travelers and the importance of adequate treatment and timely repatriation these conditions are often overlooked, underestimated, and inadequately managed, potentially leading to severe health outcomes, including prolonged hospitalization abroad potentially affecting the outcome of treatment.

There is lack of research on travel-related mental health and there is no data on how the quality of outcome, care and repatriation in those patients compares to domestic patients. Recognizing and understanding these factors is key to improving preventive strategies [15] and intervention strategies. Comprehensive knowledge of the phenomenon of travel-related psychiatric disorder would also enable mental health professionals to provide more tailored and effective treatment plans.

The aim of this study is to demographically and clinically characterize the phenomenon of travel-related mental disorders in a large patient collective and to identify group specific outcome factors of travelers by comparing them to domestic patients and migrants using propensity score matching. Due to the large catchment area of our institution in a major city with international connections, we consider our collective to be representative of the phenomenon of pathological or pathogenic travel. To the best of our knowledge there is no such data published yet.

## Methods

### Study design, data sources

The Department of Adult Psychiatry and Psychotherapy as part of the Psychiatric University Hospital of Zurich, is responsible for the psychiatric inpatient treatment of adult patients in the City of Zurich, Switzerland, and its surroundings, with a catchment area of approximately 500,000 inhabitants. Our study retrospectively analyzed electronic health records of all travelers admitted and discharged from our hospital between January 1st, 2013, and December 31st, 2020. We extracted routine clinical data from electronic health records for the present study. The Ethics Committee of the Canton of Zurich authorized the use of the anonymized data for research and publication purposes (BASEC: 2018 − 01906).

We used sociodemographic, clinical, and service use variables for the present analysis. Sociodemographic variables included age, sex, marital and educational status, German language proficiency, migration status, and country of origin. The clinical variables we used were the main treatment diagnoses according to the WHO-ICD-10 criteria; the Clinical Global Impression Scale (CGI); and the Health of the Nation Scales (HoNOS). In addition, we extracted the pharmacological and non-pharmacological treatments prescribed during the hospitalization from the clinical records. Service use variables included the type of admission, admission ward, duration of treatment, type of discharge (i.e., regular or irregular in case of discharge against medical advice, court decision, death, or suicide of inpatients), and transfer to another hospital. Travelers were defined as such if they had their permanent residence outside Switzerland and were not registered as asylum seekers. For propensity score matching each admitted traveler was matched to one Swiss national patient and to one migrant patient. Domestic migrants without Swiss nationality were selected as a separate comparison group in order to better consider factors such as a foreign home country, foreign language or cultural peculiarities as confounders. Migrants were hereby defined as people without Swiss nationality who have permanent residence in Switzerland and are legally registered and insured as such. This includes, for example, registered asylum seekers during their asylum procedure or after being granted residence status or immigrants from other countries residing in Switzerland.

In order to obtain representative and sufficiently large groups of patients, we classified the treatment diagnosis upon discharge in six diagnostic groups according to the ICD-10 categories [20]: Alcohol and Substance Use Disorder (F1) Schizophrenia Spectrum Disorders (F2), Mania and Bipolar Disorder (F30-F31), Major Depressive Disorder (F3X), Anxiety and Stress-Related Disorders (F4-F5), and Personality Disorders (F6). Furthermore, we recorded the presence of comorbid alcohol and substance use and personality disorders.

The Clinical Global Impression (CGI) Scales and the Health of the Nation Outcome Scales (HoNOS) were rated upon admission and discharge. The CGI is an easily applicable measurement instrument to assess severity (CGI-S) and improvement or deterioration during hospitalization (CGI-I). CGI-S is rated on a seven-point Likert scale from 1 (“normal”) to 7 (“extremely ill”). The CGI-I evaluates changes in comparison to the previous CGI evaluation. It ranges from 1 (“very much improved”) to 7 (“very much worse”), whereby a score of 4 indicates no change [21, 22].

The HoNOS is a measurement instrument used to assess the severity of psychiatric disorders in 12 different domains covering behavior, symptomatology, impairment, and psychosocial functioning. Each item is rated on a five-point Likert scale from 0 (“no problem”) to 4 (“severe to very severe problem”). We evaluated the HoNOS at scale level (i.e., sum score ranging from 0 to 48) and item level [23–26]. We considered HoNOS Items rated three or four as clinically significant and as an integral part of the patients’ care plan [26].

### Statistical analysis

According to the principle of independence, the analysis only included the first admission between January 1st, 2013, and December 31st, 2020. Descriptive statistics (mean, standard deviation, median, interquartile range - IQR, and percentages) were used to characterize the travelers admitted to the hospital during the observation period.

We used the propensity score to represent the probability of individual cases to be a traveler, conditional on their observed characteristics. Using logistic regression, we determined the relationship between travelers and sociodemographic and clinical characteristics, and service use patterns. Odds ratios (OR) were calculated with a 95% confidence interval (CI). Therefore, categorical variables were dichotomized, allowing to assess the risk associated with a single condition in contrast to the absence of this specific condition.

In a further step, we calculated the propensity score using logistic regression with the sociodemographic, diagnostic, clinical characteristics, service use patterns of patients upon admission. Conditional on the propensity score, the distribution of observed baseline covariates will be similar between travelers, Swiss nationals, and migrant (with a residency status) patients, allowing to assess the unbiased effect of traveler status [27].

Each admitted traveler was matched to one Swiss national patient, and to one migrant patient. Thus, leading to final 1:1:1 ratio. For the calculation of the propensity score we included demographic variables (age, sex, education, German language proficiency, marital status); provenience (German speaking country, neighbor-country, Schengen-country or other Continent of origin); clinical (diagnosis, comorbidity, clinical severity, clinical characteristics); and service use variables (type of admission; type of admission ward; time of the day at admission). This was conducted based on their nearest neighbor on the propensity score scale; with the smallest absolute, averaged propensity score distance across all included subjects [28, 29]. If no matching pair was found, cases were excluded to guarantee similar distribution of variables in the secondary dataset.

To assess the balance between the groups (before and after matching), we used the standardized mean difference (SMD) for continuous variables, the Chi-square (χ^2^) test for proportions, as well as propensity score distribution before and after matching. We conducted an equivalence test for statistically different variables with a low effect size to determine whether the observed effect was smaller than our smallest effect size of interest (SD = 0.50). We chose a half standard deviation since it is consistently considered as a minimally important difference in health outcomes [30, 31]. Two separated one-sided tests were performed to determine if the observed effect was greater than the lower bound (i.e., SD > − 0.50) and less than the upper bound (i.e., SD < + 0.50). Equivalence can be stated when the confidence interval lies within the equivalence boundaries [31–33].

All subsequent analyses were conducted with the propensity score-matched sample. Variables measured at discharge were used to estimate the differences in treatment prescribed and outcomes between compulsorily and voluntarily admitted patients. We used analysis of variance (ANOVA), with a subsequent pair wise Student’s t-test to assess differences in continuous variables and the Chi-square test (χ^2^) for differences in proportions. For changes in HoNOS sum scores, from admission to discharge, a single-factor independent group analysis of covariance (ANCOVA) was used to test for differences according to the migration status (i.e., traveler, Swiss national and migrant), thereby controlling for variability in scores upon admission. Kaplan-Meier time-to-event curves representing time to discharge (i.e., duration of treatment) was calculated; for testing the statistical significance, we use the log-rank *p*-value.

All tests of significance were two-tailed. Due to the large sample size, *p*-values less than 0.05 were considered significant. For significant results, SMD was used to evaluate effect sizes. For the analysis of the single HoNOS items, a Bonferroni correction for repeated measurements was performed. Because all remaining analyses were considered exploratory, no further correction for multiple comparisons was performed.

Statistical analyses and figures were conducted using RStudio (2024.04.0 + 402); the statistical software R (4.1.2); and the R packages: tidyverse (1.3.1), TOSTER (0.3.4), MatchIt (4.3.1), survival (v 3.2–13), and survminer (0.4.9).

## Results

### Demographic and clinical characteristics of the study population

Between January 1st, 2013, and December 31st, 2020, 324 travelers were admitted. The mean age was 40.1 (13.7) years, with 39.5% (*n* = 128) females. Low German language proficiency occurred in 42.9% (*n* = 139) of the sample. Over two thirds (68.8%, *n* = 223) were compulsory admissions. Half of the travelers had a diagnosis of schizophrenia spectrum disorder (51.2%, *n* = 166), the other half was almost equally distributed between: anxiety and stress-related disorders (13.0%, *n* = 42), bipolar disorder (12.0%, *n* = 39), substance use disorders (10.5%, *n* = 34), and major depressive disorder (9.3%, *n* = 30); with a small number of patients with a personality disorder (4.0%, *n* = 13). Upon admission, travelers had a CGI-S score of 4.97 ± 1.08; accountable as between markedly and severely ill. They had HoNOS sum score (20.65 ± 7.66); also reflecting a severe disorder. For further details see Table 1. In comparison to Swiss nationals, migrants had a lower probability of being compulsory admitted (OR 0.88 95%CI: 0.81–0.96), while travelers had a higher probability (OR 5.78 95%CI: 4.59–7.32). Accordingly, migrants had a lower probability to being admitted on a closed or facultative closed ward (OR: 0.80; 95%CI 0.74–0.87), while travelers had a higher probability (OR 3.26; 95%CI: 2.38–4.60) than Swiss nationals.

**Table 1:** Demographic and clinical characteristics of the Sample

	National	Migrant	Traveler	
	*n* = 14,672	*n* = 3446	*n* = 324	SMD
	*M (SD)*	*M (SD)*	*M (SD)*	
Age (years)	44.82 (18.40)	39.63 (14.45)	40.09 (13.67)	0.417
	*n (%)*	*n (%)*	*n (%)*	
Sex				0.224
Male	7254 (49.4)	1895 (55.0)	196 (60.5)	
Female	7418 (50.6)	1551 (45.0)	128 (39.5)	
Civil Status				0.351
Married	3126 (21.3)	1057 (30.7)	32 (9.9)	
Single	7646 (52.1)	1528 (44.3)	150 (46.3)	
Unmarried/Other	3900 (26.6)	861 (25.0)	142 (43.8)	
Education				0.455
Regular school	7469 (50.9)	1910 (55.4)	216 (66.7)	
Apprenticeship	4724 (32.2)	952 (27.6)	46 (14.2)	
College/university	2479 (16.9)	584 (16.9)	62 (19.1)	
Language Distance				1.808
German	13,762 (93.8)	701 (20.3)	122 (37.7)	
Germanic	0 (0)	174 (5.0)	33 (10.2)	
Indo- European	910 (6.2)	1592 (46.2)	114 (35.2)	
Other Language Family	0 (0)	979 (28.4)	55 (17.0)	
Language Proficiency				0.789
Low	910 (6.2)	907 (26.3)	139 (42.9)	
Geographic Distance				3.428
Swiss	14,672 (100.0)	0 (0.0)	0 (0.0)	
Austria Germany	0 (0)	712 (20.7)	123 (38.0)	
Neighbor Country	0 (0)	453 (13.1)	34 (10.5)	
Europe/Schengen	0 (0)	549 (15.9)	65 (20.0)	
Other Continent	0 (0)	1732 (50.3)	102 (31.5)	
Type of admission				0.513
Compulsive	4164 (28.4)	893 (25.9)	223 (68.8)	
Referral	5386 (36.7)	1389 (40.3)	53 (16.4)	
Walk-In	5122 (34.9)	1164 (33.8)	48 (14.8)	
Admission Ward				0.395
Facultative locked	3996 (27.2)	910 (26.4)	51 (15.7)	
Locked ward	6161 (42.0)	1306 (37.9)	233 (71.9)	
Open ward	4515 (30.8)	1230 (35.7)	40 (12.3)	
Main Diagnosis				0.638
Substance Use Disorder	2477 (16.9)	593 (17.2)	34 (10.5)	
Anxiety Disorder	2552 (17.4)	747 (21.7)	42 (13.0)	
Bipolar Disorder	1163 (7.9)	197 (5.7)	39 (12.0)	
Major Depressive Disorder	4738 (32.3)	1138 (33.0)	30 (9.3)	
Personality Disorder	1128 (7.7)	222 (6.4)	13 (4.0)	
Schizophrenia	2614 (17.8)	549 (15.9)	166 (51.2)	
Comorbid Disorder				
Substance Use	2552 (17.4)	598 (17.4)	27 (8.3)	0.113
Personality Disorder	1369 (9.3)	252 (7.3)	8 (2.5)	0.184

### Propensity score matched paired sample

Using propensity score matching, we obtained a matched sample of 972 patients in a 1:1:1 ratio between travelers, Swiss nationals and migrants. We could find a matched pair for all travelers admitted in the observation period. Mean age of the sample was 40.07 (SD = 13.99) years, with 384 (39.5%) of females. After propensity score matching there were no differences between the groups regarding the demographic or clinical characteristics. Furthermore, between the migrant and traveler groups there were no differences regarding country or region of origin.

### Treatment, clinical outcomes, and Service Use parameters

While hospitalized, the main treatment offered to travelers was crisis intervention (defined as an acute, short-term response focused on immediate stabilization, after which patients can be transitioned to appropriate follow-up care) (89.5% vs. 77.5%, χ^2^(2) = 20.74, *p* < 0.0001). Travelers were less frequently assigned to other treatments, such as individual psychotherapy (χ^2^(2) = 17.17, *p* < 0.0001) or group psychotherapy (χ^2^(2) = 14.11, *p* < 0.001), occupational therapies (χ^2^(2) = 43.34 *p* < 0.001), with slightly lower rates of counseling (47.2% vs. 40.9%, χ^2^ (1) = 36.8, *p* < 0.001), observation (χ^2^(2) = 9.02, *p* < 0.01),

There were no differences for the rate of psychopharmacological treatment (χ^2^(2) = 1.95, *p* = 0.37) in general. Antidepressants were more frequently prescribed to the migrant population (χ^2^(2) = 18.34, *p* < 0.001); while anxiolytics less frequently to the Swiss nationals (χ^2^(2) = 9.55, *p* = 0.008). There were no differences regarding the prescription of mood stabilizers (χ^2^(2) = 2.48, *p* < 0.028), antipsychotics (χ^2^(2) = 2.00, *p* < 0.37) stimulants (χ^2^(2) = 5.22, *p* = 0.07), and opioids (χ^2^(2) = 1.65, *p* = 0.043).

There were no differences regarding the use of coercive measures, either as forced medication (χ^2^(2, 972) = 1.959, *p* = 0.38) or seclusion or restraint (χ^2^(2, 972) = 2.902, *p* = 0.23). However, Swiss nationals experienced less frequent a compulsory retention (χ^2^(2, 972) = 13.183; *p* = 0.001).

At admission there was no difference in severity between the three groups CGI-S (*p* = 0.58) and HONOS scores (*p* = 0.93). At discharge travelers had a poorer CGI-I score (F(2,969) = 5.719; *p* = 0.003) than Swiss nationals and migrants. Swiss nationals had a higher HoNOS scores at discharge (F(2,969) = 8.72; *p* < 0.001); with a lower score difference between admission and discharge (F(2,969) = 3.337: *p* = 0.036) than migrants and travelers. Swiss nationals furthermore had a higher number of clinical relevant HoNOS items (F(2,969) = 6.98; *p* < 0.001) at discharge. However, there were no differences in the percentage of change (F(2,969) = 2.21:*p* = 0.11).

The HoNOS sum score improved for all groups from admission to discharge (*F*(5, 1808) = 215.0, *p* < 0.001), demonstrating that all groups experienced a significant improvement during hospitalization. Upon discharge, the HoNOS sum score (F(2,969) = 8.72; *p* < 0.001), and the number of clinically relevant items were similar (F(2,969) = 6.98; *p* < 0.001) was higher for Swiss patients; with a lower score difference between admission and discharge (F(2,969) = 4.47: *p* = 0.012). However, there were no differences in the percentage of change (F(2,969) = 2.21:*p* = 0.11). According to CGI-I, traveler patients experienced less improvement (F(2,969) = 5.72; *p* = 0.003), although absolute differences remained small. For details see Table 2.

**Table 2:** Clinical characteristics and outcome of the propensity score matched sample

	National	Migrant	Traveler			
	*n* = 324	*n* = 324	*n* = 324	Statistic	*p*	SMD
	*M (SD)*	*M (SD)*	*M (SD)*			
Admission						
CGI-S	4.71 (1.03)	4.75 (1.07)	4.97 (1.08)	*F*(2, 969) = 0.543	0.58	0.052
HoNOS	20.18 (8.16)	20.34 (8.16)	20.65 (7.66)	*F*(2, 969) = 0.071	0.93	0.020
HoNOS (Items > 3)	4.13 (2.28)	4.23 (2.47)	4.22 (2.32)	*F*(2, 969) = 0.164	0.85	0.028
Discharge						
CGI-I	2.46 (0.98)	2.43 (0.90)	2.66 (0.98)	*F*(2, 969) = 5.719	0.003	0.163
HoNOS	12.01 (6.85)	10.50 (6.62)	9.98 (5.82)	*F*(2, 969) = 8.718	< 0.001	0.210
HoNOS (Items > 3)	1.48 (2.05)	1.18 (1.95)	0.91 (1.79)	*F*(2, 969) = 6.983	0.001	0.196
HoNOS Difference	9.55 (7.96)	10.84 (8.59)	11.19 (8.07)	*F*(2, 969) = 3.337	0.036	0.134
HoNOS Percentage	41.92 (33.63)	45.76 (36.62)	47.86 (35.34)	*F*(2, 969) = 2.212	0.11	0.113
	*n (%)*	*n (%)*	*n (%)*			
Compulsive Measures						
Medication	35 (10.8)	29 (9.0)	40 (12.3)	*X*^2^(2,972) = 1.959	0.38	0.077
Restraint	32 (9.9)	27 (8.3)	40 (12.3)	*X*^2^(2,972) = 2.902	0.23	0.078
Service Use Parameters						
Length of Stay	24.74 (28.62)	24.69 (29.42)	9.69 (14.31)	*F*(2, 969) = 38.743	< 0.001	0.451
Retention	21 (6.5)	50 (15.4)	39 (12.0)	*X*^2^(2,972) = 13.183	0.001	0.177
Regular Discharge	305 (94.1)	300 (92.6)	300 (92.6)	*X*^2^(2,972) = 0.801	0.66	0.062
Transfer (Hospital)	25 (7.7)	26 (8.0)	79 (29.4)	*X*^2^(2,972) = 50.85	< 0.001	0.311

**Fig. 1:**
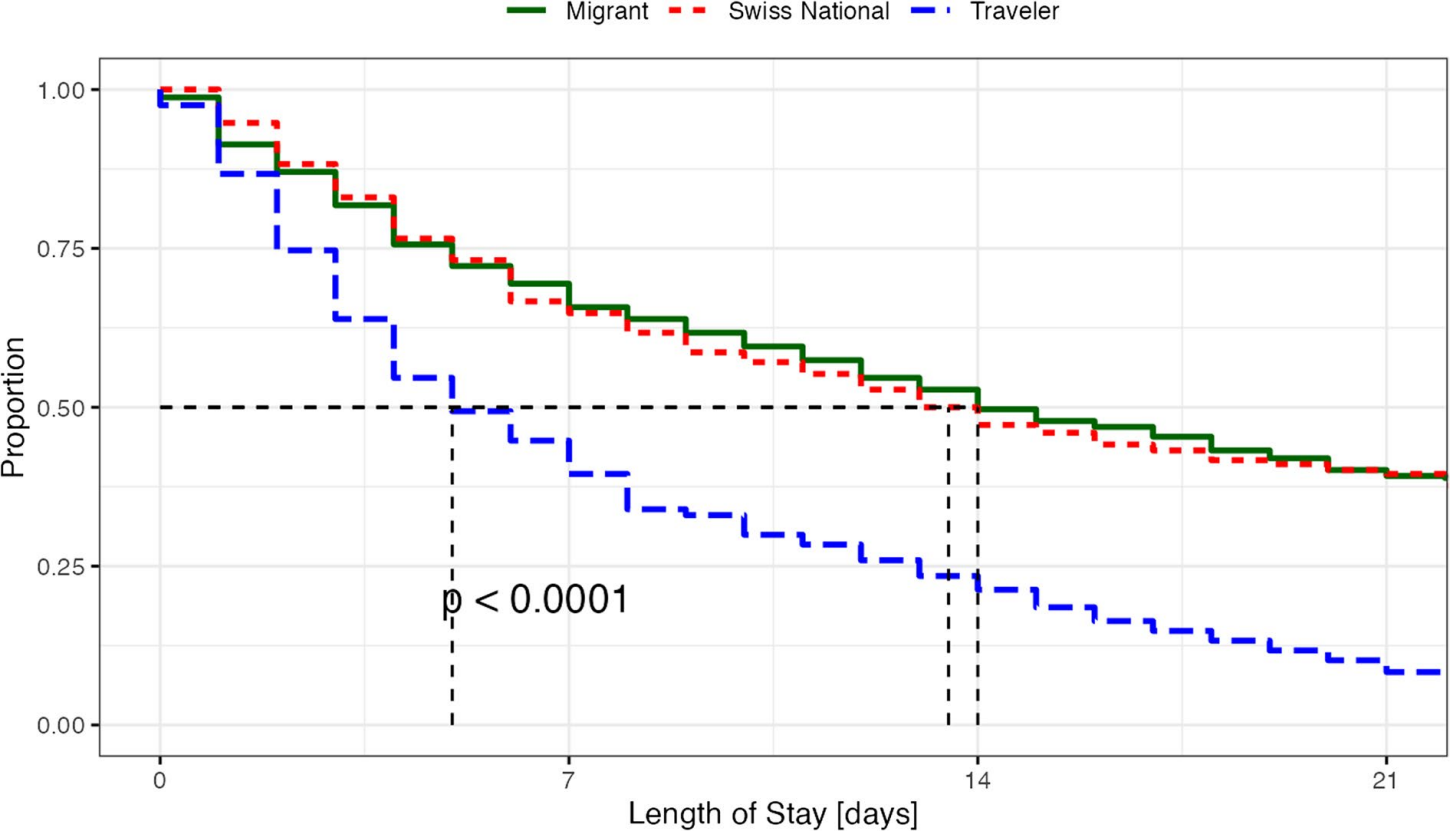
Length of Stay according to migration status. Kaplan-Meier curve depicting the probability of hospital discharge (length of stay) (y-axis) over time (x-axis)

 The length of stay was shorter (*F*(2,969) = 38.74:*p* < 0.001) for travelers (9.69 ± 14.31) than for Swiss nationals (24.69 ± 29.42) and migrants (24.74 ± 28.62). For all three groups the length of stay was right-skewed for travelers (median 5; IQR: 11); Swiss nationals (median: 13.5; IQR: 31) and migrants (median: 14.0; IQR: 28). See also Fig. 1. There were no differences regarding the rate of regular medical discharge between the groups (χ^2^(2) = 1.27;*p* = 0.53). Travelers had higher transfer rates (*X*^2^(2,972) = 50.85: *p* < 0.001) to another hospital (79, 29.4%), than Swiss nationals (25, 7.7%) or migrants (26, 8.0%). We analyzed the length of stay according to the main diagnosis (*F*(5,964) = 9.20 :*p* < 0.001); patients with substance use disorder and anxiety disorder hat a shorter length of stay than those with bipolar disorder, schizophrenia and major depression. Finally, we analyzed the length of stay according to the geographic distance of the country of origin of the travelers (*F*(2,321) = 3.11; *p* = 0.5) without finding any difference; there were also no differences regarding the distribution of treatment diagnoses (*F*(5,318) = 1.47: *p* = 0.2).

## Discussion

Our analysis utilizing propensity score matching yielded a balanced comparison between travelers, Swiss nationals, and migrants, revealing significant differences in hospitalization patterns, treatment protocols, and clinical outcomes across these groups. While all patients were provided with psychiatric care during hospitalization, the treatment modalities differed between groups. Travelers were primarily offered crisis intervention, with similar rates of pharmacological treatment, while less frequent psychotherapy (either in individual or group sessions) and occupational therapies. This could be attributed to the transitory nature of travelers’ stay, necessitating swift emergency interventions to stabilize their conditions.

Overall, schizophrenia is significantly overrepresented among travelers at over 50% compared to the other two groups. This supports the theories that patients with pre-existing psychotic disorders are more vulnerable to travel-related exacerbations (pathogenic influence of travel) or travel due to the psychotic disorder itself (pathological travel). This observation aligns with previously described data [7].

Psychopharmacological treatment was largely consistent across groups, barring a few differences. Migrants were more likely to be prescribed antidepressants, while Swiss nationals had a lower frequency of anxiolytics prescriptions. These variations may reflect differing patterns of psychiatric disorders or responses to treatment across these populations. However, we found no distinct pattern for the treatment of travelers. The use of coercive measures, such as forced medication or seclusion and restraint, were comparable across groups. However, Swiss nationals were less likely to undergo compulsory retention, suggesting potential cultural, legal, or systemic differences influencing treatment approaches. The higher rate of compulsory admissions among travelers has already been described in the Swiss healthcare system [34, 35]. A possible reason for higher rates of compulsory admission and retention presumably due to the fact that travelers are hospitalized almost exclusively in emergency situations which could be associated with a higher level of lack of insight or risk aspects. The inability to activate social resources (which might have been available when at home) when travelling may also account for the higher rates of compulsory admission and retention in travelers.

Disparities in clinical outcomes were particularly evident at discharge. Travelers had a poorer Clinical Global Impression-Improvement (CGI-I) score at discharge, indicative of a less favorable response to treatment compared to Swiss nationals and migrants. This suggests that while the overall condition of travelers might have been more severe, the degree of improvement was less substantial compared to the other two groups. Despite these disparities, all groups showed significant improvement from admission to discharge, as demonstrated by a decrease in HoNOS sum score, illustrating the effectiveness of the psychiatric interventions provided. However, travelers had a significantly shorter length of stay in the hospital compared to the other two groups. This may reflect the transient nature of tourism, necessitating expedited discharge processes or potentially shorter periods of intensive treatment which is limited to the extent of an emergency treatment and is often continued in the country of origin after repatriation [18].

For our analysis we included exclusively travelers, defined as people who have their permanent resident outside Switzerland. Other, fluctuating populations like refugees were excluded since their “living and legal” situation is far more complex and dependent on other variables [36]. Regular medical discharge rates were similar across the groups, indicating comparable end-of-treatment conditions despite differences in length of stay. We considered the transfers to another hospital for travelers as proxy for repatriation, considering that our institution has a referral status (i.e. all diagnostic and treatment possibilities are available) the main and sometimes solely indication for the transfer to another hospital is to allow close to home treatment. Finally, the length of stay appeared to be influenced by the main diagnosis implying potential roles of disease severity.

In summary, as travelers tend to be admitted in emergency situations associated with higher rates of compulsory admission it has to be ensured that travelers receive adequate care and are not prematurely discharged, potentially leading to risks to self or others or worsening conditions upon return to their home country. This should include training for healthcare providers in particular in tourist-heavy regions to recognize and manage psychiatric crises in travelers including repatriation at the earliest point in time after effective emergency treatment as recommended in current literature [18]. This includes cultural competence training to deal with a diverse patient population effectively to ensure that repatriation is done safely and ethically. International guidelines that standardize psychiatric repatriation to enhance patient outcomes would hereby be useful as well as international standards on coverage of costs of repatriation.

There are some limitations that need to be considered. The design of the study is retrospective. There is no follow-up data on the outcome of travelers. Outcome measurement is restricted to general scales like GCI or HoNOS due to the lack of standardized diagnose-specific outcome measurement in our data. Cultural background of travelers remains unclear. Furthermore, since our sample data were derived from routine clinical practice thus the demographic and clinical details differ from those obtained in controlled trials [37]. In contrast, the requirement for hospitalization underlines the disabling nature of the disorder [38, 39]. Some travelers had health insurance that covered emergency treatment, for example due to bilateral agreements (European Union) or private insurance, while other travelers did not. The exact insurance status was not analyzed, so its influence on treatment cannot be mapped. We considered the transfer to another hospital as a proxy for repatriation, however if and were the patients continued treatment is unknown. The reported length of stay in our study must be interpreted with caution, as it only reflects the duration of hospitalization in our facility and does not account for potential continued psychiatric inpatient treatment after repatriation to the patient’s home country.

Overall, this study is the first to uncover the nuances in psychiatric care and outcomes among travelers compared to groups of domestic patients emphasizing the importance of understanding these differences to tailor interventions effectively and enhance psychiatric care for diverse patient populations. Inpatient treatments of travelers have shown to be shorter in general presenting a more severe overall condition at discharge. Travelers were admitted involuntarily more frequently. This most closely reflects the theory that travelers are hospitalized only in clinically serious emergency situations and are discharged or repatriated as soon as possible after the completion of effective emergency treatment. Further studies should focus on travel-related mental health as there still is a lack of knowledge in this field.

The findings of this study have important clinical implications for psychiatric care of travelers. Given their higher rates of compulsory admission, shorter lengths of stay, and inferior clinical improvement scores at discharge compared to domestic patients, clinicians should implement a structured approach including: rapid diagnostic assessment, intensive crisis intervention focused on immediate stabilization, and early planning of repatriation including communication with healthcare providers in the patient’s home country to ensure continuity of care. Particular attention should be paid to patients with schizophrenia spectrum disorders, as they represent over 50% of traveling patients and may be especially vulnerable to travel-related exacerbations. Emergency psychiatric services, particularly in tourist-heavy regions, should develop standardized protocols for the assessment and management of traveling psychiatric patients.

## Data Availability

No datasets were generated or analysed during the current study.

## Abbreviations

HoNOS: Health of the Nation Outcome Scale
ICD-10: International Statistical Classification of Diseases and Related Health Problems, 10^th^ revision
SD: Standard Deviation
